# The Role of Uncertainty and Negative Emotion in Chinese Parents’ Self-Medication of Children with Antibiotics

**DOI:** 10.3390/ijerph20166603

**Published:** 2023-08-18

**Authors:** Di Pei, Gary Kreps, Xiaoquan Zhao

**Affiliations:** 1 School of Public Health, Georgia State University, Atlanta, GA 30302, USA; dpei@gsu.edu; 2 Department of Communication, George Mason University, Fairfax, VA 22030, USA

**Keywords:** self-medication, antibiotics, parental caregiving, China

## Abstract

Parents self-medicating their children with antibiotics (SMA) without consulting healthcare professionals is a common practice in China. Previous research has focused primarily on examining the socioeconomic factors that contribute to the prevalence of SMA. This study adopts and extends the theory of planned behavior to understand the cognitive and emotional factors that are associated with parental SMA in China. The responses to an online survey were collected from 961 parents of children aged 6–12 years old, primarily from Gansu, Shandong, and Shaanxi provinces. More than half of the participants (66.5%) engaged in parental SMA. Amoxicillin, Cephradine, and Azithromycin were the most frequently selected antibiotics used for children. Structural equation modeling showed that uncertainty was positively associated with negative emotions, which were in turn positively associated with attitude toward SMA. Uncertainty was also negatively associated with perceived behavioral control (PBC), but the association between PBC and SMA behavior was not significant. Attitude and subjective norm were both positively associated with SMA behavior. The relationship between subjective norm and attitude was also positive. Understanding the psychological factors driving parental SMA may inform tailored interventions to promote responsible antibiotic use among parents.

## 1. Introduction

Self-medication is a common self-care practice. It is defined as “the use of medicinal products by the consumer to treat self-recognized disorders or symptoms, or the intermittent or continued use of a medication prescribed by a physician for chronic or recurring diseases or symptoms” [1]. Responsible self-medication can promote a long, healthy life, and many healthcare programs have claimed that appropriate self-care and self-medication should be the first step of effective treatment [1,2]. However, inappropriate self-mediation can lead to many health risks, such as delayed diagnosis and treatment, undue therapy, pathogen resistance, and drug interactions [1,3]. Among the potential risks, antibiotic resistance is identified as one of the biggest challenges to global public health [4].

As a powerful treatment for bacterial infections, antibiotics have saved millions of lives since they were discovered. But antibiotics also break the balance of our microbiome system and destroy both the bacteria that are causing the illness and those that protect us against infections. It has been widely acknowledged that the misuse of antibiotics might cause many fatal side effects, including the development of multidrug-resistant pathogens within the human body. A recent report in *The Lancet* suggests that antibiotic resistance caused an estimated 1.27 million deaths and was associated with about 4.95 million deaths worldwide in 2019 [5].

Self-medication with antibiotics (SMA) has been particularly common in low- and middle-income countries (LMICs). The unique combination of factors in these regions, such as less developed health systems, limited supervision and regulation, and reduced adherence to medical guidelines, create an environment conducive to the prevalence of this issue [6,7]. A systematic review indicates that the prevalence of SMA in LMICs ranges from 8.1% to 93% [6]. Emerging research also suggests that the outbreak of the COVID-19 pandemic may increase the risks of antibiotic resistance among LMIC residents, where antibiotics are often overused or misused to treat or prevent COVID-19 infections [8,9].

An emerging trend in China is parental self-medication for children [10,11,12,13]. Young children are at a vulnerable age and are not able to make informed decisions about how to manage their own health. Parents and caregivers often medicate children based on superficial interpretations of their symptoms, and without consulting any health professionals. The misuse of antibiotics in children can be particularly problematic. Early-life antibiotic use might cause atopic disorders (allergic problems such as dermatitis, rhinitis, and asthma) among children [14]. The use of antibiotics in infancy and childhood is associated with an increased occurrence of respiratory infections and the development of asthma in children who are predisposed to atopic immune responses [15].

The trend of antibiotics misuse by parents in China clearly calls for more in-depth investigations and interventions. While the existing literature on parental SMA focuses primarily on examining the scale of the issue and the socioeconomic factors that contribute to its prevalence [10,11,12,13], a better understanding of the psychological factors contributing to the prevalence of parental SMA in China is essential for developing effective tailored interventions for Chinese parents. These interventions can play a pivotal role in equipping parents with the knowledge and psychological support necessary for making informed medical decisions. Such initiatives could help curb the inappropriate use of antibiotics and foster a culture of effective self-care practice, thereby promoting better health outcomes for children in China. To address the gap and contribute to the literature, the current study uses the theory of planned behavior as a guiding framework to examine the cognitive and emotional determinants that affect parents’ medical decision-making regarding antibiotic use for their children.

### 1.1. Parental Self-Medication with Antibiotics in China

The prevalence of parental SMA in China is drawing increasing attention in recent years. According to a meta-analysis, 37.8% of Chinese caregivers have self-medicated their children with antibiotics at home [16]. Other studies revealed that living in rural areas, having more than one child, an older child age [17], lower income [18], and previous self-medication experiences [19] are primary risk factors for Chinese parents’ SMA practices with their children. Common illnesses that often lead to parental SMA include colds, sore throats, and fevers [13].

Although national strategies and regulations have been put forward to regulate the sale of antibiotics through prescriptions and to promote the rational use of antibiotics across the country [20], challenges have arisen in implementing the plan. Due to relatively loose regulations and limitations in resources and staff, consumers in small cities and rural areas still have easy access to antibiotics. A 2019 study conducted in the Zhejiang, Hubei, and Sichuan provinces in China indicated that 73.3% of visitors to community pharmacies successfully obtained antibiotics without a doctor’s prescription [21]. Moreover, Chinese parents reported that purchasing antibiotics from community pharmacies is one of their main sources for getting non-prescription antibiotics [19]. Another main source of access to antibiotics for parents is through storing leftover medicines at home. Research shows that over 48.1% of Chinese parents keep leftover antibiotics at home for future use [13].

### 1.2. The Theory of Planned Behavior

The theory of planned behavior (TPB) suggests that people’s intention to perform a certain behavior is determined by their attitude, subjective norm, and perceived behavioral control over the behavior [22]. Furthermore, behavioral intention and perceived behavioral control are directly linked to behavioral performance. According to the TPB, attitude is an individual’s subjective assessment of the desirability of a behavior. Subjective norms refer to the perceived social pressure from important others to perform specific behaviors. Perceived behavioral control represents an individual’s perceived capability in performing the behavior.

**Figure 1:**
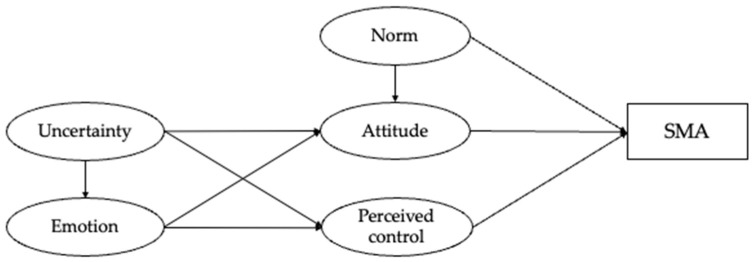
A hypothesized conceptual model based on an extended theory of planned behavior.

The TPB has been used to explain and predict a wide range of health-related behaviors, including self- medication without consulting healthcare professionals [23,24,25]. Overall, the findings were largely consistent across the contexts, indicating that attitude, subjective norm, and perceived behavioral control are significantly associated with people’s self-medication intentions and behaviors [26,27,28,29]. However, the extent to which the TPB can help explain parental SMA for children in China is unknown. A better understanding of the cognitive and emotional factors related to parents’ medical decision-making for their children may provide important insights for future education and regulation aimed at addressing antibiotic misuse in children. Based on the TPB, the present study hypothesizes that parents’ attitudes toward SMA for children, the extent to which they think important others approve of the use of antibiotics for children, and their perceived ability to perform SMA for children are positively associated with their SMA for children. These hypothesized relationships are presented in a conceptual model in Figure 1.

### 1.3. The Relationship between Subjective Norm and Attitude in the Chinese Cultural Context

The Chinese cultural context exhibits a particularly strong connection between social norms and the initiation of health behaviors. In a country that greatly values harmony and collectivism, people are more likely to adapt their attitudes and behaviors to conform with others than those in individualistic cultures [30,31,32]. The perceived social norm can affect individuals’ attitudinal evaluations of the desirability of a behavior [33,34,35]. Previous studies have tested the TPB on various behavior domains in China [36,37,38,39] and other cultural contexts [40,41,42,43], and have found that subjective norms can directly influence people’s attitude towards the behavior, which subsequently affects their intentions and performance of the behavior. Building on this established connection between subjective norm and attitude in the Chinese context, the present study hypothesizes that Chinese parents’ perceived norm for using antibiotics with children will influence their attitude towards parental SMA.

### 1.4. Parents’ Uncertainty and Negative Emotions during Children’s Illness

To further enhance the explanatory power of the TPB in various contexts, researchers have proposed additional constructs that could be integrated into the theory, including uncertainty and emotions. Uncertainty is a common experience when people are dealing with illness. Mishel’s theory of uncertainty in illness defines uncertainty as the inability to determine the meaning of illness-related events [44]. According to Mishel, uncertainty in illness consists of four dimensions [45,46]. First, people with an illness face ambiguity related to the state of the illness. The symptoms of diseases often generate ambiguity through the difficulty of interpreting them and identifying accurate causes. Second, people face the complexities of treatment and systems of care. Uncertainty arises when people are unable to determine the effectiveness of the treatment or have difficulty understanding treatment instructions. The third dimension of uncertainty in illness concerns deficient information regarding the diagnosis and the seriousness of the condition. The last dimension involves the unpredictability of the course of the illness. The presence of one or more of these dimensions indicates heightened uncertainty during illness. 

As children’s primary caregivers, parents often experience uncertainty concerning the causes, courses, and outcomes of children’s illness [47]. When parents experience uncertainty, their ability to appraise the situation and make optimal decisions is limited [48]. Parental uncertainty in children’s illness is positively related to parents’ psychological distress, including anxiety, depression, cognitive disturbance, perceived helplessness [49,50], and financial burden [49], as well as parents’ impaired cognitive capacity and misjudgments of children’ illness [45,51,52]. 

Previous research suggests uncertainty as an antecedent construct that predicts one’s attitude, subjective norm, and perceived behavioral control over the behavior, which, in turn, predicts one’s behavioral intention [53,54]. In the context of parental SMA for children, it is likely that the increased uncertainty during children’s illness could impact parents’ medical decision-making for their children. We hypothesize that parents’ uncertainty in children’s illness is an antecedent variable that is associated with attitudes and perceived behavioral control over self-medicating children with antibiotics, which could, in turn, influence their actual SMA behavior.

Effective care for children with an illness is dependent on parents’ ability to make rational medical decisions based on sensible cognitive appraisals and a calm mental state. However, during children’s illness, parents often experience increased levels of negative emotional reactions, including anxiety, fear, anger, and loneliness [55,56,57]. Previous research has found that the negative emotions experienced by parents influence the outcome of caregiving. For example, parents who were taking care of children with a mental illness found it difficult to take care of their own mental well-being and maintain a sense of order and structure [58]. Parents of children with substance abuse disorders reported that the emotional stress coming from feelings of worry and sadness outweighed their perceived financial stress [59]. Neuroscientific research shows that negative emotions, such as anxious and stressful feelings, could impair people’s ability to share knowledge and the quality of their decision-making [60]. There is also evidence that caregivers’ negative emotions could compromise recovery and health outcomes [61,62]. The present study, therefore, proposes that parents’ negative emotions during children’s illness will be positively associated with their attitude towards and perceived ability to self-medicate children with antibiotics, which will, in turn, influence their SMA behavior. 

As suggested in previous studies [49,50], parents’ uncertainty regarding their children’s illness is expected to influence their negative emotional reactions, such that stronger uncertainty is associated with more negative emotions. No directional relationship was hypothesized between uncertainty and subjective norm because parents’ assessment of the social norms of using antibiotics for their children is likely to be stable, and unlikely to be affected by the increased uncertainty or negative emotions during children’s illness [53,54]. All the hypothesized relationships are presented in Figure 1.

## 2. Methods

### 2.1. Study Design

An online survey was conducted with Chinese parents of children aged 6–12 years old living in three provinces in China: Gansu (northwest), Shandong (northeast), and Shaanxi (central area). Previous studies suggest that the age of the child is positively associated with parental SMA in China [17]. We selected primary school-age students as our study population as they heavily rely on their parents for medical decision-making, may have lower health literacy than their older peers, and are more likely to be self-medicated than younger children. Participants were recruited through local primary schools and social media groups or forums where parents of school children tend to congregate. 

The survey was developed and administered through the Qualtrics online survey platform. Participants accessed the survey by clicking on the posted link, and they provided consent online before proceeding to complete demographic questions and respond to questions assessing the main variables. The study was approved by the George Mason University Institutional Review Board.

### 2.2. Measures

The main variables were measured using established scales that have been used and validated in many research studies spanning various disciplines. More information about these measurement instruments is provided below. The survey questions are presented in Appendix A. 

#### 2.2.1. Parental Uncertainty in Children’s Illness

Six items selected from the Mishel Uncertainty in Illness, Family Member Form were modified to measure Chinese parents’ uncertainty regarding children’s illness [44,46]. To adapt to the present study, the concept “children’s illness” was added as a boundary condition for the statements. Sample items included: “It is unclear to me how I should take care of my child”; “My child’s symptoms continue to change unpredictably”. Statements were rated on a 5-point Likert scale, from 1, strongly disagree, to 5, strongly agree.

#### 2.2.2. Parental Negative Emotional Reactions to Children’s Illness

Five questions from the Brief Illness Perception Scale were adapted to measure the extent to which parents experience negative emotions in response to children’s illness [63]. On a scale from 1, not at all, to 7, very, they were asked to rate their feelings of anger, fear, sadness, and anxiety, and their depression levels during children’s illness.

#### 2.2.3. Attitude towards SMA for Children

Three items were used to measure parents’ attitudes towards SMA for children [54,64] on a scale from 1, strongly disagree, to 5, strongly agree, such as “Antibiotics will help my child recover sooner”.

#### 2.2.4. Subjective Norm

Parents’ perceived social pressure to practice SMA on children was measured using six items [54,64], such as: “My family members think I should use antibiotics for my child when she/he is sick”; “Most parents I know think I should use antibiotics to treat children’s illness”. Parents rated their level of agreement with these statements on a scale from 1, strongly disagree, to 5, strongly agree.

#### 2.2.5. Perceived Behavioral Control over Medicating Children with Antibiotics

Three items from Coleman and Karraker’s Self-efficacy for Parenting Tasks Index, the Health Subscale were modified to measure parents’ perceived control over using antibiotics for children [65]. Parents rated their level of agreement on a scale from 1, strongly disagree, to 5, strongly agree, with the statements, including: “I am able to make the right medical decision for my child”; “I can use the antibiotic medicine to improve his/her condition”.

#### 2.2.6. SMA Behavior

Parents’ SMA behavior was measured with a single item: “In the past year, how many times did you use antibiotics for your child without prescriptions?” Parents provided the actual number of times they had engaged in SMA (M =2.39, SD = 0.87).

### 2.3. Statistical Analysis

After computing the descriptive analyses, the conceptual model was tested using structural equation modeling (SEM). The three variables from the theory of planned behavior (attitude, norm, and perceived behavioral control), as well as the two added variables (uncertainty and negative emotions), were all specified as latent constructs with multiple observed items serving as indicators. An overall confirmatory factor analysis model (CFA) including all the latent variables was first estimated to examine the measurement adequacy. The CFA allowed us to assess the relationships between the observed indicators and their respective latent constructs, providing evidence for the construct validity of our measurements. By analyzing the goodness-of-fit indices and factor loadings, we could verify the appropriateness of the scale used to measure each latent variable. After testing the measurement model, full structural paths were added and estimated. The analysis was conducted using PROC CALIS in SAS. The model fit was evaluated using the comparative fit index (CFI) and root mean square error of approximation (RMSEA), with a CFI of 0.95 or higher and an RMSEA of 0.06 or lower considered as indicating good fit [66].

## 3. Results

### 3.1. Descriptive Statistics

More than 1000 respondents completed the online survey. Prior to the analysis, the data were examined for incomplete responses and respondents who had more than 40% of unanswered questions were removed. The data were also examined for univariate normality. The frequency distribution of the main variables approximated a bell-shaped curve. A total of 961 responses were retained for data analysis. The existing literature suggests 10–20 subjects per estimated parameter [67]. Our sample size surpassed the criteria, providing adequate statistical power for a model estimation. Among the respondents, 289 were from Shandong province (30.1%), 207 were from Gansu (44.2%), 425 were from Shaanxi (21.5%), and 30 were from other regions of China (0.3%). The majority of the respondents were mothers (70.7%), 27.9% were fathers, and 1.4% were other family members who self-identified as children’s primary caregivers. The mean age of the respondents was 36.65 (SD = 4.44), and 9.16 for their children (SD = 2.15). Descriptive statistics of respondents’ demographic information are presented in Table 1.

### 3.2. Antibiotic Use for Children

Overall, 735 (76.5%) of the parents in the study had self-medicated their children in their lifetime. Most respondents (*n* = 568, 66.5%) had used antibiotic medicines at least once during the year prior to the survey (Table 1). The percentages of performing parental SMA for children differed significantly among parents with different family incomes and among parents living in different provinces. Table 2 presents the different types of antibiotics that were commonly used for children. Amoxicillin was found to be the most frequently used antibiotic drug among Chinese parents (*n* = 521, 54.2%), followed by Cephradine (*n* = 300, 31.2%), and Azithromycin (*n* = 243, 25.3%). Parents were more likely to self-medicate their children instead of consulting a doctor when observing their children having the symptoms of a runny nose (M =2.63, SD = 1.26), nasal congestion (M = 2.68, SD = 1.28), and a sore throat (M = 3.01, SD = 1.30).

### 3.3. SEM Analysis of the Conceptual Model

The CFA model had an adequate fit, with X^2^ (179) = 500.63, *p* < 0.001, CFI = 0.95 and RMSEA = 0.05. All the items were significantly loaded on the corresponding latent variables, with item loadings ranging from 0.45 to 0.90. We then proceeded to test the full conceptual model.

The SEM model examining the hypothesized associations was estimated. Path coefficients are presented in Table 3. To control for the effects of demographic differences, demographic variables were allowed to influence both exogenous and endogenous variables. Only the age of the child and family income were significantly associated with some of the variables and were kept in the final model. The model allowed for the covariation of norm and perceived behavioral control, as well as covariances of uncertainty and norm, negative emotion and norm, and perceived behavioral control and attitude. The full SEM model fits the data well, with X^2^ (229) =534.59, *p* < 0.001, CFI = 0.95, and RMSEA = 0.04. Overall, the model accounted for 22%, 23%, 11%, and 12% of the variance in Chinese parents’ SMA behavior, attitude, perceived behavioral control, and negative emotion, respectively.

**Figure 2:**
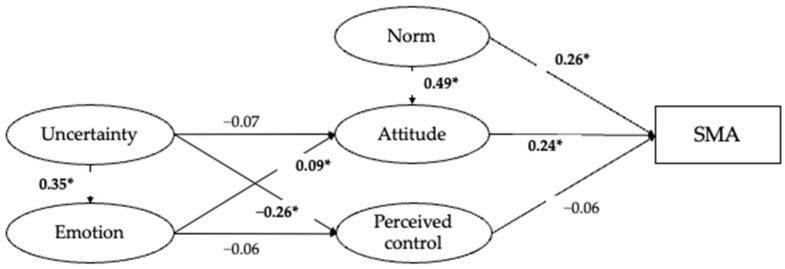
Path coefficients in the full SEM model. Note: * indicates significance at *p* < 0.05.

As illustrated in Figure 2, parents’ perceived uncertainty was significantly associated with their negative emotional reactions to children’s illness (β = 0.35, *p* < 0.001), indicating that higher perceived uncertainty was related to more negative emotions. Furthermore, the more negative emotions parents experience during children’s illness, the more positive attitudes they have toward self-medicating with antibiotics for their children (β = 0.09, *p* < 0.05). Meanwhile, the more parents feel uncertainty during children’s illness, the less perceived control they have over performing SMA for children (β = −0.26, *p* < 0.001).

Among the TPB variables, the subjective norm of performing SMA for children was significantly associated with parents’ attitude towards SMA for children (β = 0.49, *p* < 0.001), which suggests that parents who perceive stronger social pressure to practice SMA for their children tend to have a more positive attitude towards SMA for children.

Both attitude (β = 0.24, *p* < 0.001) and subjective norm (β = 0.26, *p* < 0.001) were significantly associated with parents’ actual SMA behavior. Specifically, the more social pressure parents perceive regarding performing SMA for children, the more likely they would be to self-medicate their children with antibiotics. Meanwhile, the more positive attitudes parents hold towards practicing SMA on children, the more likely they would be to perform SMA. Perceived behavioral control, however, was not significantly associated with parental SMA for children (β = −0.06, *p* = 0.09).

## 4. Discussion

More than half of the surveyed parents (66.5%) reported using antibiotics for their children without consulting healthcare professionals. This percentage is higher than in previous research conducted in China (37.8%) [16], as well as research investigating the issue in other LMICs (ranging from 3.7% to 44.0%) [68]. Amoxicillin, Cephradine, and Azithromycin were the most frequently selected antibiotics that Chinese parents used for their children. The results indicate that parental self-medication with antibiotics remains a prevalent practice among Chinese parents.

Both uncertainty and negative emotions are important factors to consider in explaining parental SMA for children in China. In line with the four dimensions of uncertainty people have in illness suggested by Mishel’s theory of uncertainty in illness [45,46], Chinese parents experience ambiguity in figuring out how to provide care for children and assess the effectiveness of medications; complexity in interpreting symptoms; lack of information when choosing from different treatment options; and unpredictability in terms of the course and duration of the illness. The increased uncertainty influences their negative emotional reactions to children’s illness, which subsequently affects their medical decision-making.

The results supported the hypothesis that Chinese parents’ uncertainty in children’s illness is positively associated with their negative emotional reactions. This finding is consistent with evidence documented in other contexts that examine the relationship between uncertainty and emotions [49,50]. When Chinese parents feel uncertain, confused, and ambiguous about how to make optimal medical decisions for their sick children, they are more likely to experience elevated anxiety, anger, fear, sadness, and depression. These uncertain and negative emotional experiences can impair parents’ cognitive capacity and lead to misjudgments about their children’s illness [45,69]. As shown in the final model, negative emotion was positively associated with parents’ attitude towards SMA for children. Specifically, when parents experience more negative emotions during their children’s illness, they tend to have a more positive attitude towards using antibiotics with their children. This increased positive attitude, in turn, is linked to an increased likelihood of self-medicating their children with antibiotics.

Subjective norm appeared to be an important factor influencing parental SMA in China. As shown in the final model, subjective norm was significantly associated with both parents’ attitudes toward SMA for children and their actual SMA behavior. This finding is expected, given the strong connection between social norms and the initiation of health behaviors in the Chinese cultural context [36,37,38,39]. In cultures where collectivism and harmony are valued, individuals tend to conform to the social norms and may experience feelings of guilt when these norms are violated [70]. Chinese people are likely to make behavioral changes based on their beliefs about others’ perceptions and expectations [71,72]. In the present study, when parents believed important others in their family and social groups approve of using antibiotics with children, they tended to have more positive attitudes towards SMA behavior. As hypothesized, subjective norm is also directly linked to parental SMA behavior, with greater perceived social pressure to use antibiotics for children indicating a higher likelihood of making the SMA decision.

The assumption that uncertainty is associated with perceived behavioral control [54,73,74] was supported in the study. Higher levels of uncertainty were associated with weaker beliefs in one’s ability to use antibiotics to treat children’s illnesses. When parents face ambiguous and unpredictable situations related to their children’s illness, their ability to make rational and informed decisions is compromised, leading to a perceived lack of control over effectively medicating their children. However, our study did not find a significant association between parents’ perceived behavioral control and SMA behavior. One possible explanation for this is that normative factors, such as subjective norm, tend to have a stronger impact than self-evaluative factors in the specific cultural context of China. Chinese parents’ beliefs about the treatment advice from their social groups, combined with their increased positive attitude towards SMA, might have significantly influenced their SMA decision, while the self-evaluation of their ability to use antibiotics to treat children’s illness may have been less important.

### 4.1. Theoretical and Practical Implications

Our study contributes to the literature on parental SMA by applying the theory of planned behavior as a guiding framework to help understand the cognitive and emotional factors underlying this practice in China. Our study extends previous research by examining uncertainty and negative emotions as potential antecedents of the TPB variables. Significant associations were found between uncertainty and perceived behavioral control, and between negative emotions and attitude. These additional constructs were shown to have a significant impact on parents’ attitudes toward self-medicating children with antibiotics, which ultimately influences their decision to engage in SMA.

Additionally, our findings highlight the importance of social norms in understanding health behaviors in the Chinese context. In line with previous research exploring the association between attitude and subjective norm, our findings also suggest that subjective norm may not only directly affect parental SMA behavior in China, but also indirectly influence behavior through parents’ attitudes toward SMA for children. Future studies employing the TPB framework may consider exploring the interrelationships among the TPB constructs as they may offer further insights into health decision-making across various domains of behavior.

Based on the findings, we have several recommendations for future health education and interventions aimed at reducing parental antibiotic misuse in China. First, it is important to promote parents’ cognitive appraisal of uncertain situations, such as children’s illness. Health educational campaigns can work with local hospitals and communities to provide more informational and social support, which may help to increase parents’ knowledge and skills in effectively interpreting the causes and symptoms of children’s illness and reduce parents’ perceived uncertainty and negative emotional reactions in these unexpected situations. Next, accurate information on self-medication, especially the use of antibiotics, needs to be disseminated to the public. Developing effective health communication messages may help parents understand the potential risks of antibiotic misuse at both an individual and societal level. Last, changing the perceived social norms regarding antibiotic use may be an effective approach. Creating a normative environment where the self-initiated use of antibiotics in children is less common and not socially acceptable may help reduce parental antibiotics misuse and make parents more cautious when it comes to self-medication for children in general.

### 4.2. Limitations

This study has limitations that should be acknowledged. First, this was a cross-sectional survey; thus, the associations identified should not be directly interpreted as causal. A longitudinal study would be necessary to establish the causal pathways among the main variables. Second, the study used self-reported data, which is subject to social desirability and recall bias. However, considering the survey respondents might want to please the researcher by under-reporting rather than over-reporting their antibiotic use, the explained variance in parental SMA is likely to be suppressed rather than exaggerated. Finally, most of the measurements used in the study were adopted from scales that were developed in the U.S., which might have limited applicability to the Chinese context.

## 5. Conclusions

Chinese parents’ perceived uncertainty was positively associated with their negative emotional reactions to children’s illness, which was, in turn, positively associated with their attitude towards SMA for children and their SMA behavior. Subjective norm played an essential role in explaining parents’ SMA decisions for children in the China context. Health education and interventions are needed to increase parents’ knowledge and skills in providing care for ill children and to reduce their uncertainty and negative emotions during children’s illness. Creating a normative environment where parental SMA is less common and not socially acceptable may help to reduce parental antibiotics misuse in China.

## Figures and Tables

**Table 1:** Sample characteristics and patterns of parental self-medication with antibiotics.

Sample Characteristics	Valid *n*	Percentage % (95% CI)	*n* of SMA Behavior in a Year (Continuous) ^1^ Mean (95% CI)	Ever Performed SMA in a Year ^2^ (Dichotomous) % (95% CI)	*p* Value
Overall	961	100%	2.4 (2.3–2.4)	66.4 (63.24–69.62)	
Relation to children	947				*p* = 0.25
Mother	670	70.7 (67.8–73.7)	2.5 (2.4–2.6)	67.7 (63.9–71.4)	
Father	264	27.9	2.3 (2.3–2.4)	62.6 (56.3–68.9)	
Other	13	1.4	2.3 (1.8–2.9)	80.0 (65.2–94.8)	
Age (years)	950				*p* = 0.38
18–29	20	2.1 (1.2–3.0)	2.8 (2.4–3.2)	63.2 (41.4–84.9)	
30–39	695	73.2 (70.3–76.0)	2.4 (2.3–2.4)	67.0 (63.3–70.7)	
40–49	227	23.9 (21.2–26.6)	2.3 (2.2–2.5)	65.0 (58.3–71.6)	
50+	8	0.8 (0.3–1.4)	2.3 (1.6–3.0)	66.7 (45.2–88.2)	
Children’s Age (years)	950				*p* = 0.37
6–9	526	55.4 (52.2–58.5)	2.4 (2.4–2.5)	65.2 (60.8–69.5)	
9–12	424	44.6 (41.5–47.8)	2.3 (2.3–2.4)	68.1 (63.4–72.8)	
Family Monthly Income (RMB)	951				*p* = 0.006 *
3000 or less	124	13.0 (10.9–15.2)	2.0 (1.9–2.2)	72.7 (64.4–81.1)	
3001–7000	414	43.5 (40.4–46.7)	2.7 (2.6–2.9)	70.5 (65.9–75.2)	
7001–13,000	278	29.2 (26.3–32.1)	2.4 (2.3–2.5)	64.4 (58.4–70.4)	
13,001–60,000	126	13.2 (11.1–15.4)	2.7 (2.1–3.2)	53.9 (44.8–63.0)	
More than 60,000	9	0.9 (0.3–1.6)	2.4 (2.3–2.5)	17.7 (15.3–85.7)	
Education level	949				*p* = 0.51
Less than high school	113	11.9 (9.8–14.0)	2.5 (2.3–2.7)	62.5 (53.2–71.8)	
High school	205	21.6 (19.0–24.2)	2.4 (2.3–2.5)	66.1 (59.2–73.0)	
Vocational school	240	25.3 (22.5–28.1)	2.3 (2.2–2.4)	63.9 (57.5–70.3)	
College	274	28.9 (26.0–31.8)	2.3 (2.2–2.5)	69.0 (63.2–74.8)	
Higher than college	117	12.3 (10.2–14.4)	2.5 (2.3–2.6)	71.6 (62.8–80.3)	
Residential Area (Province)	951				*p* = 0.001 *
Shandong	289	30.1 (27.2–33.0)	2.6 (2.5–2.7)	58.5 (52.7–64.3)	
Gansu	425	44.2 (41.1–47.4)	2.5 (2.2–2.8)	73.2 (68.6–77.7)	
Shaanxi	207	21.5 (18.9–24.1)	2.1 (2.0–2.2)	68.8 (61.7–75.5)	
Other	40	4.2 (2.9–5.4)	2.4 (2.2–2.5)	46.4 (27.9–64.9)	

Note: ^1^ *n* of SMA behavior in a year = the actual number of times participants had engaged in SMA in a year; ^2^ Ever performed SMA in a year was dichotomized as “0 = never performed SMA for children in the past year” and “1 = have performed SMA for children at least once in the past year”. * Statistically significant (*p* < 0.05) based on the Rao-Scott Chi-squared test.

**Table 2:** Types of antibiotics used by parents for their children.

Types of Antibiosis	*n*	% (95% CI)
Amoxicillin	521	54.2 (51.1–57.4)
Cephalothin	300	31.2 (28.3–34.2)
Azithromycin	243	25.3 (22.5–28.0)
Cefalexin	180	18.7 (16.3–21.2)
Erythromycin	120	12.5 (10.4–14.6)
Norfloxacin	76	7.9 (6.2–9.6)
Penicillin	68	7.1 (5.5–8.7)
Streptomycin	59	6.1 (4.6–7.7)
Levofloxacin	52	5.4 (4.0–6.8)
Chloramphenicol	3	0.3 (0–0.7)
Other	110	11.4 (9.4–13.5)

**Table 3:** Path coefficients in the full SEM model.

Path from	Path to	β	SE	*p*
Uncertainty	Negative emotion	**0.35**	0.04	<0.001
Uncertainty	Attitude	−0.07	0.05	0.16
Uncertainty	Perceived control	**−0.26**	0.04	<0.001
Negative emotion	Attitude	**0.09**	0.04	0.04
Negative emotion	Perceived control	−0.06	0.04	0.13
Social norm	Attitude	**0.49**	0.04	<0.001
Social norm	SMA	**0.26**	0.04	<0.001
Attitude	SMA	**0.24**	0.05	<0.001
Perceived control	SMA	−0.06	0.04	0.09

Note: Bold indicates significance at *p* < 0.05.

## Data Availability

The data presented in this study are available on request from the corresponding author.

## Appendix A

Survey Questions

1. Parental uncertainty in children’s illness

Think about a situation when you are taking care of your child with illness. How much do you agree with the following statements? (1—strongly disagree, 5—strongly agree)

It is unclear to me how I should take care of my child.

Effectiveness of the medicines is undetermined.

My child’s symptoms continue to change unpredictably.

My child’s symptoms were too complex for me to figure out what was wrong.

I have been told different things about what to do to take care of my child.

I have a lot of questions without answers.

2. Parental emotional reactions to children’s illness

How much do you experience the following emotions when your child is ill?

1—Not at all angry, 7—Very angry.

1—Not at all scared, 7—Very scared.

1—Not at all upset, 7—Very upset.

1—Not at all depressed, 7—Very depressed.

1—Not at all anxious, 7—Very anxious.

3. Attitude towards SMA for children

Think about the conditions when you want to use antibiotics for your child without a doctor’s guidance. How much do you agree with the following statements? (1—strongly disagree, 5—strongly agree)

Antibiotics will help treat the disease completely.

Antibiotics will help my child recover sooner.

Antibiotics will protect my child from further infections and severe diseases.

4. Subjective norms about SMA

How much do you agree with the following statements regarding your friends and family’s attitudes toward antibiotic use? (1—strongly disagree, 5—strongly agree)

Most parents in my social network would self-medicate for their children.

I feel more comfortable using antibiotics for my child if others in my social network recommend it to me.

I feel more comfortable using antibiotics for my child if others in my family agree with my medical decision.

Others in my family think I should use antibiotic medicines for my child when he/she is ill.

5. Perceived behavioral control

Think about the conditions when you want to use antibiotics for your child without a doctor’s guidance. How much do you agree with the following statements? (1—strongly disagree, 5—strongly agree)

I am able to make the right medical decisions for my child.

My medical treatment can improve his/her condition.

My medical treatment can minimize the negative influence of his/her illness.

6. Parental SMA for children

In the past year, how many times did you use antibiotics for your child without prescriptions?
